# Opinion: Yes

**DOI:** 10.1590/S1677-5538.IBJU.2016.04.02

**Published:** 2016

**Authors:** Caio Cesar Cintra, Carlos Eduardo Bonafé Oliveira

**Affiliations:** 1 Departamento de Urologia, Faculdade de Medicina do ABC, SP, Brasil

**Keywords:** Suburethral Slings, Surgical Mesh, Pelvic Floor, Prolapse

Nowadays, synthetic meshes are widely used in reconstructive surgeries of the pelvic floor. However, since the publication of the FDA warning about associated complications in 2011 (1), several discussions and contrary opinions have been published about its usefulness. The fear of court lawsuits related to side effects, common in some settings, has contributed to the widening of global discussion.

Doubtless, these slings are associated to specific complications, such as exposition and erosion, and impact on sexual performance of treated patients. However, the big question is: is the use of meshes in pelvic surgery always problematic?

- The use of mesh to treat prolapses is equal to its use in stress urinary incontinence?
- Is the use of these slings in the correction of prolapses of different grades and positions the same?

In other words, is it possible to expand the complications rates from one indication to others, and vice-versa?

When we evaluate the history of the use of slings in reconstructive surgery of the pelvic floor, this reasoning of generalization was used since the beginning. Slings were introduced as a minimal invasive procedure without the need of incisions to reach healthy tissues, with good results and low rate of complications at long follow up. The use of meshes was widened to include the treatment of pelvic prolapses with a valid theoretic reasoning that conventional surgeries showed high rates of recurrence (2). This fact corresponded to the beginning of use of the same materials in large scale.

Urologists and gynecologists started to progressively employ these meshes frequently with low training. After some years, the complications emerged. Publication of results of the use of meshes in the treatment of pelvic prolapses by several authors justified the positioning of FDA (1).

In that document, the agency cited 10% of exposition/erosion of meshes in patients treated for pelvic prolapses inserting the mesh vaginally after 12 months of surgery (3). However, in some studies, this rate was even higher, reaching almost 33% (4).

On the other hand, the incidence of erosion/exposition of mesh in the abdominal correction of prolapses is inferior, around 4%, in a follow up of 23 months (5), implying that the access way and not only the use of meshes is related to the high level of observed complications.

Equally, erosion rate of midurethral slings is 2%, according to FDA. Only 3 to 5% of patients evaluated by the TOMUS trial presented complications related to meshes in a follow up of 24 months (6).

In relation to sexual performance, 6.2% of women submitted to sling surgery presented dyspareunia, in a 3 year-follow up study (7). After treatment of prolapses via vaginal appliance of meshes, dyspareunia was referred by 24.4% of patients (8), emphasizing again different rates of the same disturbance according to different indications of their use.

Many of the complications related to the use of slings are not related to where they are applied. Pain, dyspareunia, recurrence, urinary infection and hematomas are also observed in conventional vaginal surgeries with native tissue. This fact was also stressed by SUFU in relation to the FDA warning (9).

In a comparative study, Nieminem et al showed a lower rate of dyspareunia in patients submitted to correction of prolapse with mesh, when compared to women submitted to conventional treatment (10). They confirmed that this complain is not exclusive of women treated with synthetic slings.

Therefore, should we make the same old mistake and generalize indications and side effects of prolapse correction and retrograde slings as was for the initial indications?

We don't think so, and other expert groups also endorse this position. American Urogynecologic Society (AUS) defended FDA positioning, but restricted it only to the use of meshes for correction of prolapses via vagina. AUS reinforced that such arguments should not be applied to slings or to the use of meshes via abdominal for the correction of prolapses (11).

Midurethral synthetic slings are still the gold standard technique for the treatment of stress urinary incontinence in women. EAU recommends them as first choice for non-complicated stress urinary incontinence in women (12). AUA guidelines describe the use of synthetic slings as an option for the treatment of stress urinary incontinence and recommends the discussion of specific complications with the patients as well as the benefits of quick recovery (13).

Therefore, in our point of view, it is equivocal to condemn in general the use of midurethral slings via vagina. General fear should not mask reality, the years of experience and published data.

As physicians, we should endorse the best available evidences. Although the significant number of complications related to the treatment of prolapses justify precaution, slings should be viewed as good alternatives, with acceptable side effects, inferior to those observed with the use of meshes via vagina to correct prolapses.

According to good practice standards, we should guide and obtain signed consent of the patients, similar to any surgical procedure involving the use of prosthesis, reinforcing that side effects are specific for this kind of treatment.
